# The downstream PPARγ target LRRC1 participates in early stage adipocytic differentiation

**DOI:** 10.1007/s11010-022-04609-8

**Published:** 2022-11-12

**Authors:** Xinping Wang, Jianyun Liu, Ting Wang, Baicheng Ma, Ping Wu, Xiaoyuan Xu, Jianjun Xiong

**Affiliations:** College of Basic Medical Science, Jiujiang University, 551 Qianjindong Road, Jiujiang, 332005 China

**Keywords:** LRRC1, PPARγ, Mesenchymal stem cells (MSCs), Adipocytic differentiation

## Abstract

LRRC1 is a regulator of cellular polarity that is expressed at high levels in a range of tumor tissue types. Here, we conducted an analysis of the previously unexplored role of LRRC1 as a component of the adipogenic differentiation network. During the early stage (days 3–7) adipocytic differentiation of human mesenchymal stem cells (MSCs), LRRC1 was found to be upregulated at both the mRNA and protein levels. Moreover, the expression of LRRC1 was found to be controlled by PPARγ, which is a key transcriptional regulator of adipogenesis. Inhibiting LRRC1 expression reduced the adipogenic potential of hMSCs, with a concomitant reduction in the expression of three adipogenesis-associated proteins (SCD, LIPE, FASN). Together, these data offer new insight into the functional importance of LRRC1 both in general and in the context of adipocytic differentiation.

**Supplementary Information:**

The online version contains supplementary material available at 10.1007/s11010-022-04609-8.

## Introduction

Adipose tissue is of key physiological importance in the context of energy homeostasis, the maintenance of an appropriate body temperature, and the physiology and pathology of a range of organ types [1]. Adipocytic differentiation is thus an area of active research interest. Adipocytes originate from bone marrow-derived mesenchymal stem cells (MSCs) through a complex two-stage adipogenic process that consists of the initial development of MSC-derived lineage-committed preadipocytes followed by the full differentiation and maturation of these preadipocytes to yield functional adipocytes [2]. A number of transcriptional changes are associated with the adipogenic process, with transcription factors including peroxisome proliferator-activated receptor (PPARs) and CCAAT/enhancer-binding proteins (C/EBPs) being integral to this overall process [3, 4]. Downstream targets of these transcription factors include MDM2, TAF7L, and ZNF638, which can form a complex network that regulates cellular differentiation [5–7].

In a previous study, we utilized next-generation sequencing to identify changes in mRNA expression profiles that occur during the early stages of adipocytic differentiation [8], with this approach revealing LRRC1 (leucine-rich repeat-containing 1, gene ID: 55227) to be upregulated in these differentiating cells. LRRC1 is encoded on chromosome 6p12.3 and p12.2 in humans and is reportedly expressed in renal, prostate, pancreatic, placental, colon, thyroid, and adrenergic gland tissue [9]. Structurally, LRRC1 consists of 524 amino acids including 16 leucine-rich repeats and a LAP-specific domain [9]. LRRC1 interacts with PDZ domain-containing proteins such as DLG1 CASK, MPP7, and SNX27, and thus participates in the homeostasis of epithelial tissues and tumor growth [10]. Functionally, LRRC1 has been linked to metastatic progression in a range of malignancies including breast and liver cancer [11, 12]. When overexpressed, LRRC1 reportedly enhanced hepatocellular carcinoma (HCC) cell growth and clonogenic activity, whereas its knockout had the opposite effect [12]. Moreover, LRRC1 enhancement has been shown to facilitate the transformation of NIH3T3 cells [12]. How LRCC1 regulates adipogenic differentiation, however, has yet to be established.

Here, we utilized primary cultured MSCs to explore the expression and physiological importance of LRRC1 in the context of adipocytic differentiation. Overall, our results both offer new insight regarding the biological importance of LRRC1 and also clarify the mechanisms governing the differentiation of adipocytes.

## Materials and methods

### Cell culture and transduction

Primary cultured MSCs were obtained from a healthy male donor and cultured as per a previously published protocol [13]. The characterization of primary MSCs was performed via flow cytometry (Supplementary Fig. 1). Adipogenesis was induced by stimulating cells from the 6th passage with an adipogenic cocktail consisting of minimum Eagle’s medium-alpha containing 10% FBS, 1.0 μM dexamethasone, 0.5 mM 3-isobutyl-1-methylxanthine, and 0.01 mg/ml insulin (Sigma, MO, USA) for 3, 7, or 14 days [14]. To knock down the expression of specific target genes (PPARγ and LRRC1) in these MSCs, specific lentiviruses encoding shRNA constructs were constructed by Shanghai Genechem Co., Ltd. Cells were then infected with these particles or control particles based upon provided directions. Briefly, cells (5 × 10^5^) were cultured overnight in a 25 cm^2^ flask, after which 5 μL of lentiviral particles were added with polybrene and incubated for 12 h, after which media was exchanged for fresh complete optimal medium. At 48 h post-transduction, media was changed and cells were used for downstream analyses. The shRNA target sequences used for PPARγ and LRRC1 are listed in Supplementary Table 1.

### RT-qPCR

SYBR Green was used to measure target gene expression via RT-qPCR. Briefly, TRIzol (Invitrogen) was used to extract RNA from cells, with cDNA then being prepared with a ReverTra Ace^®^ qPCR RT Kit (Toyobo, Osaka, Japan). A 7500 ABI instrument (ABI, CA, USA) was used for subsequent RT-qPCR analyses using the primers listed in Supplementary Table 1.

### Western blotting

Whole-cell lysates were separated via 10% SDS-PAGE and transferred onto PVDF membranes (Bio-Rad, CA, USA). Blots were blocked overnight at 4 °C in 5% non-fat milk, followed by incubation at room temperature with appropriate antibodies for 2 h. Antibodies utilized included anti-LRRC1 (1:1000, ab127568, Abcam), anti-PPARγ (1:1500, ab272718, Abcam), anti-CEBP/β (1:1000, ab53138, Abcam), anti-FASN (1:1000, ab128870, Abcam), anti-LIPE (1:1000, ab45422, Abcam), anti-SCD1 (1:1000, ab236868, Abcam), and anti-β-actin (1:100, ab6276, Abcam). Secondary HRP-conjugated antibodies (1:10,000) were used to detect protein bands, which were then detected via enhanced chemiluminescence system and analyzed with the Image Lab software.

### Chromatin immunoprecipitation (ChIP)

An EZ-ChIP Kit (Millipore) was used to conduct all ChIP assays based on provided directions. Briefly, cells were initially fixed at room temperature for 30 min with 1% formaldehyde under mild shaking, after which cells were lysed and ultrasonicated to fragment chromatin to 500–1500 bp in length on average. Samples were then centrifuged and incubated overnight with 3 mg of anti-PPARγ (Abcam, ab233218) or control IgG at 4 °C to conduct immunoprecipitation. Magnetic protein-G beads were then added and samples were incubated for 1 h at 4 °C. Samples were then washed, antibody-transcription factor-DNA complexes were eluted from DNA, formaldehyde cross-linking was reversed, and proteinase K was used to treat samples overnight at 67 °C to digest residual protein. DNA was purified and used for Real-time PCR with designed primers (Supplementary Table 1).

### Oil red O staining

On day 14 of adipogenic differentiation, cells were rinsed two times using PBS, fixed with formaldehyde, washed for 5 min using 60% isopropyl alcohol, dried at room temperature, and then stained with oil red O (1 mL/well) for 20 min. Cells were then washed two times and assessed via light microscopy. Cells were then dried at room temperature, and oil red O was eluted by adding 100% isopropanol to each well, with the absorbance of the eluted solution being measured at 490 nm [15].

### Proteomic analyses

At 48 h after LRRC1 knockdown, adipogenesis was induced and cells were collected on days 0 or 7 for proteomic sequencing performed by Wuhan SpecAlly Life Technology Co., Ltd, China. LC-MS/MS data acquisition was conducted with a Q Exactive HF-X mass spectrometer coupled to an Ultimate 3000 system. Raw MS data were analyzed using MaxQuant (V1.6.6) with the Andromeda database search algorithm. Spectra files were searched against the Swissprot human protein database. After annotating all proteins identified via this approach, relevant details for differentially expressed proteins were extracted and the STRING database (https://string-db.org/) was used to perform a protein–protein interaction analysis.

### Statistical analyses

Data were analyzed with GraphPad Prism 7.0 and are presented as the mean ± SEM. Results were compared between two groups using Student’s *t*-tests. *P* < 0.05.

## Results

### LRRC1 is upregulated during the early stages of MSC adipocytic differentiation

**Fig. 1:**
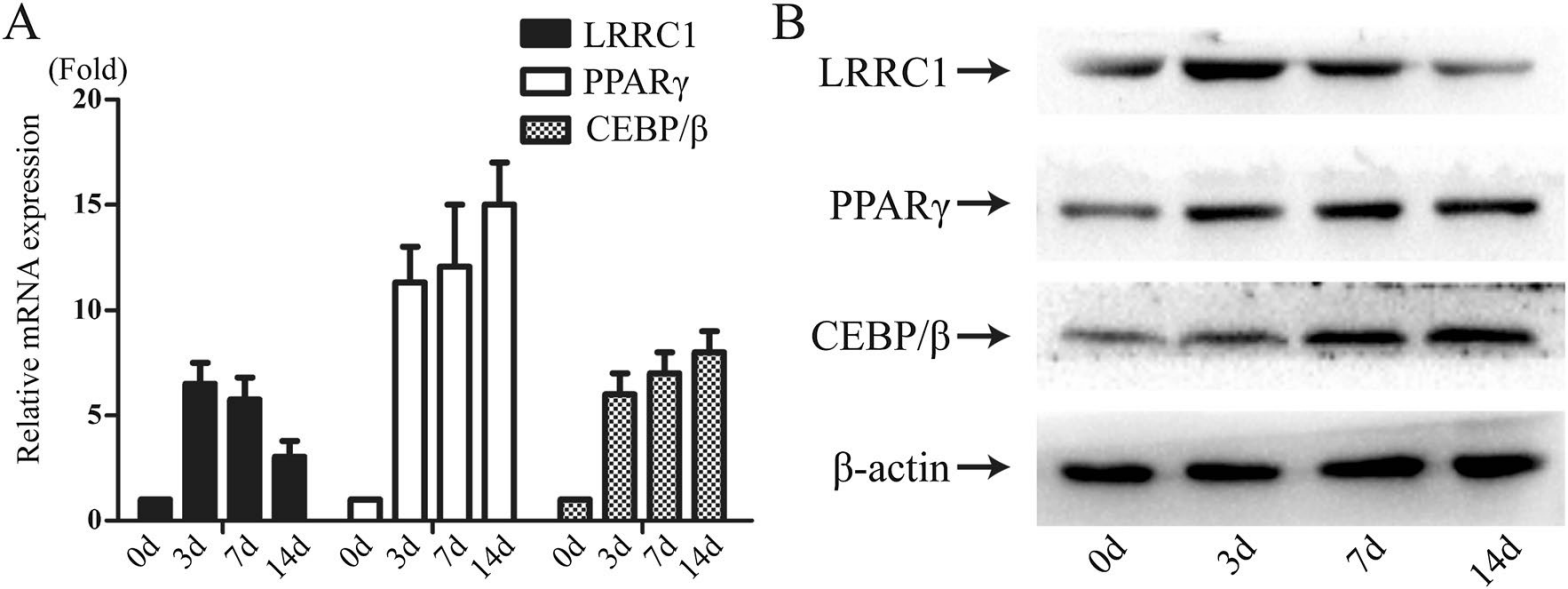
LRRC1 is dynamically expressed during MSCs adipogenic differentiation. **A** LRRC1, PPARγ, and C/EBP-β mRNA expression during the adipogenic differentiation of MSCs; **B** LRRC1, PPARγ, and C/EBP-β protein expression during the adipogenic differentiation of MSCs

Initially, adipocytic differentiation was induced in MSCs for 0–14 days, with *LRRC1* mRNA expression being assessed on days 0, 3, 7, and 14 via RT-qPCR. This analysis revealed significant *LRRC1* upregulation on day 3 (~ 6.4-fold vs. day 0) and day 7 (~ 5.5-fold vs. day 0), whereas its expression was increased by only ~ 2.8-fold on day 14 relative to day 0. The expression of the key adipogenesis-related transcription factors PPARγ and C/EBP-β was also assessed as a positive control, revealing that while all three of these genes were progressively upregulated on days 3 and 7 of adipogenesis, *LRRC1* expression was no longer synchronized with that of PPARγ and C/EBP-β on day 14 (Fig. 1A). Western blotting further confirmed these results (Fig. 1B).

### PPARγ regulates LRRC1 transcription

**Fig. 2:**
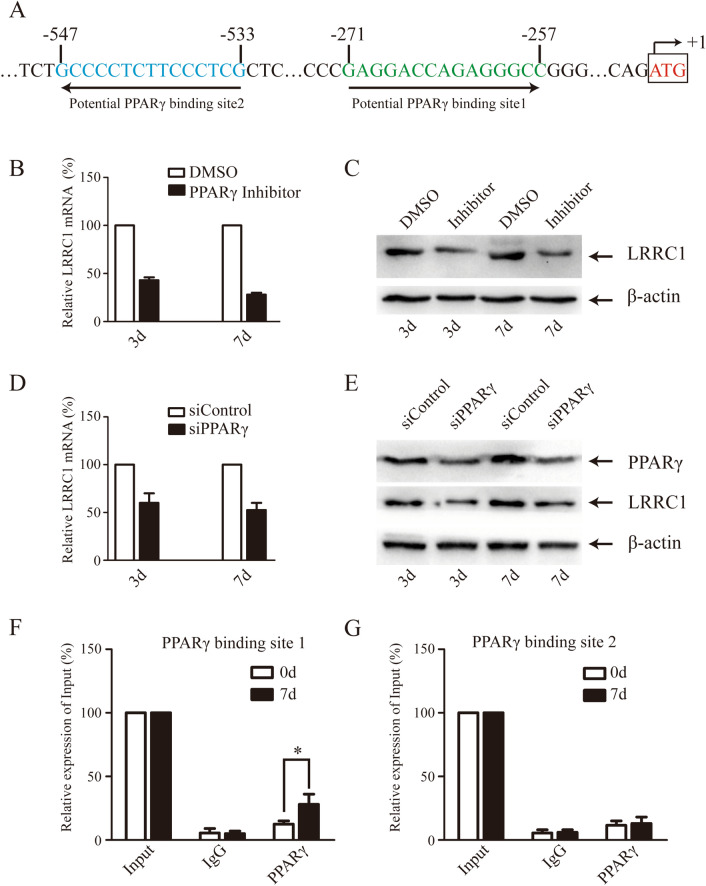
PPARγ regulates LRRC1 transcriptional activity. **A** The predicted two PPARγ binding sites adjacent translation initiation site in the LRRC1 promoter region; **B** and **C** PPARγ-specific inhibitors downregulated LRRC1 mRNA and protein expression on the 3rd and 7th day of adipogenic differentiation; **D** and **E** lentivirus mediated shRNA inhibited the expression of PPARγ, thus decreasing LRRC1 mRNA and protein expression; **F** and **G** ChIP analyses revealed significantly enhanced PPARγ binding to potential binding site 1 in the LRRC1 promoter on the 7th day of adipogenic differentiation, whereas no such binding was observed for potential binding site 2. *p < 0.05

Given the similarities in the transcriptional profiles of LRRC1, PPARγ, and C/EBP-β during the early stages of adipocytic differentiation, we next sought to identify potential binding sites for these transcription factors within the *LRRC1* promoter. Using the JASPAR database (https://jaspar.genereg.net/), we identified two putative PPARγ binding sites within 1000 bp upstream of the *LRRC1* translational starting site, including one from − 257 to − 271 (forward) and one from − 533 to − 547 (reverse) (Fig. 2A). We thus specifically explored the ability of PPARγ to regulate LRRC1 expression. When hMSCs were treated with a 10 μM dose of a PPARγ-specific inhibitor (Selleck, T0070907), significant reductions in LRRC1 mRNA and protein levels were observed on days 3 and 7 of adipogenic differentiation (Fig. 2B, C). To expand on these results, we used lentivirally delivered shRNA constructs to knock down PPARγ expression, resulting in similar reductions in LRRC1 mRNA and protein levels on days 3 and 7 of adipogenic differentiation (Fig. 2D, E). As transfection efficiency for MSCs is very low, we were unable to construct an LRRC1 promoter reporter construct for use in a luciferase-based reporter assay. However, we did assess PPARγ binding to the LRRC1 promoter in a ChIP assay which revealed significant PPARγ binding to binding site 1 (− 257 to − 271) within the LRRC1 promoter on day 7 of adipogenesis relative to day 0, whereas only limited binding to binding site 2 was observed at either of these time points (Fig. 2F, G). Together, these results suggest that PPARγ can regulate *LRRC1* transcription via binding to an upstream region within the *LRRC1* promoter.

### LRRC1 is involved in the progression of adipocytic differentiation

**Fig. 3:**
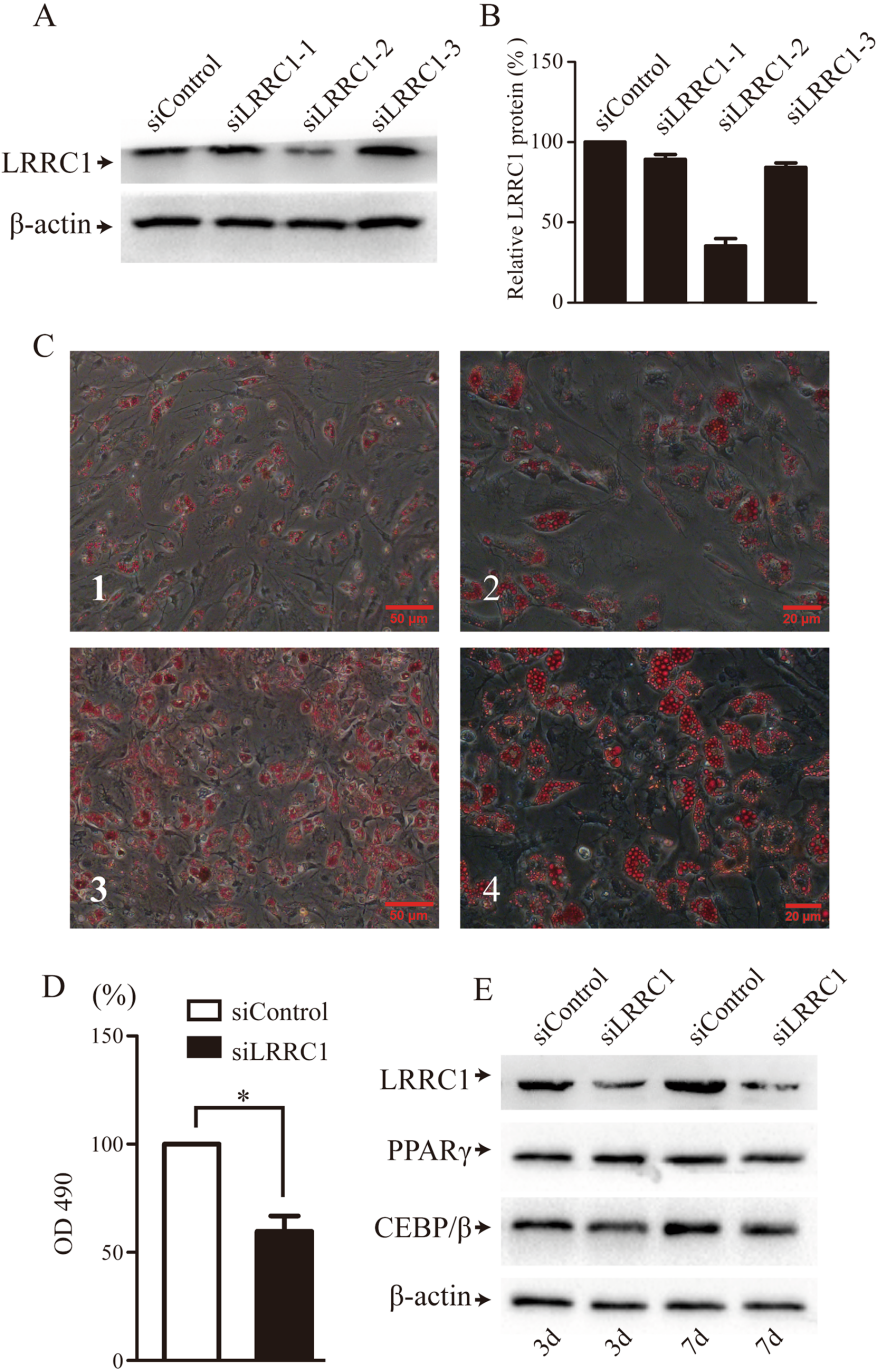
The downregulation of LRRC1 expression affects adipogenic differentiation. **A** and **B** Lentivirus-mediated shRNA expression inhibited LRRC1 expression; **C** 1–2 after inhibiting the expression of LRRC1, lipid droplets were stained with oil red O on the 7th and 14th days of adipogenic differentiation; **C** 3–4 oil red O staining was performed using negative control virus-infected MSCs at the same time points; **D** after 14 days of adipogenic differentiation, a quantitative analysis of lipid droplets revealed that lipid droplet levels were decreased in the LRRC1 knockdown group; **E** Western blotting revealed that the downregulation of LRRC1 failed to reduce the levels of the key adipogenic transcription factors PPARγ and C/EBP-β

Lenvirally mediated shRNA delivery was next used to effectively knock down LRRC1 (Fig. 3A, B). LRRC1 knockdown had no effect on MSC proliferation within 7 days after transfection (data not shown). At 48 h after lentiviral transduction, adipogenic differentiation was induced in these MSCs for 14 days, after which oil red O staining was conducted revealing that LRRC1 knockdown suppressed MSC adipogenic activity. Specifically, the numbers of lipid droplets and overall fat content were reduced in the LRRC1-knockdown group relative to the control group (Fig. 3C, D). However, LRRC1 knockdown had no impact on the expression of PPARγ or C/EBP-β (Fig. 3E).

#### LRRC1 impacts fat metabolism-related gene expression

**Fig. 4:**
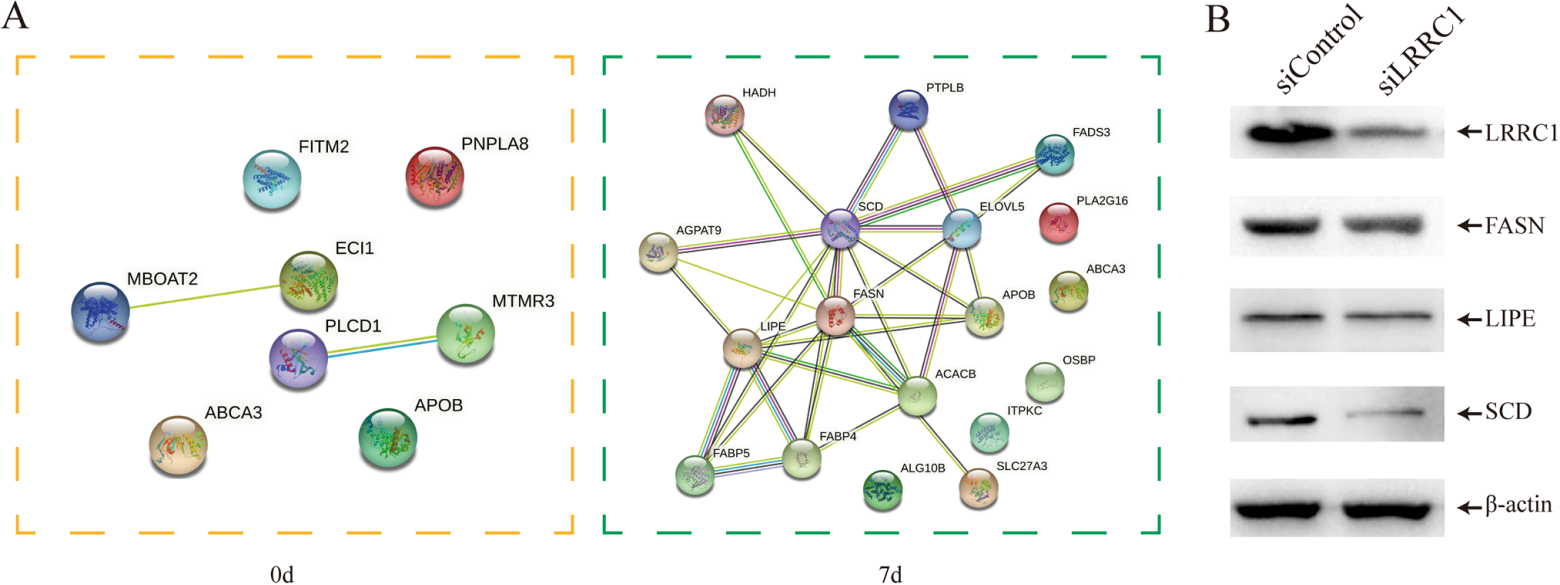
LRRC1 affects the expression of genes related to fat metabolism. **A** Fat metabolism-related genes decreased by LRRC1 inhibition were analyzed by PPI using the STRING database; **B** three representative fat metabolism-related genes were detected by western blotting on the 7th day of adipogenic differentiation

To identify downstream targets of LRRC1, we next conducted a proteomic analysis of control cells and LRRC1-knockdown cells. Using 1.2-fold as the expression threshold, 582 differentially expressed proteins were identified between the LRRC1-knockdown and control groups (210 upregulated, 372 downregulated) on day 9. On day 7 of differentiation, there were 562 differentially expressed proteins between these two groups (283 upregulated, 280 downregulated) (Supplementary Table 2). We next specifically focused on fat metabolism-related proteins. In total, 8 fat metabolism-associated proteins were downregulated in the LRRC1-knockdown group relative to the control group, and this number had risen to 18 by day 7 of differentiation (Table 1). A protein–protein interaction analysis revealed complex functional correlations among these proteins on day 7 of differentiation (Fig. 4A). Western blotting was then used to validate the changes in the FASN, LIPE, and SCD protein levels, confirming that all three were downregulated on day 7 in LRRC1-knockdown cells, in line with our proteomic results (Fig. 4B).

**Table 1:** Fat metabolism-related genes decreased by LRRC1 inhibition

LRRC1 knock down/negative control on 0 day		LRRC1 knock down/negative control on 7 day	
Gene (Accession No.)	Relative expression of negative control	Gene (Accession No.)	Relative expression of negative control
APOB (P04114)	0.735	ACACB (O00763)	0.710
ECI1 (P42126)	0.802	SCD (O00767)^a^	0.576
PLCD1 (P51178)	0.816	APOB (P04114)	0.760
MTMR3 (Q13615)	0.810	FABP4 (P15090)	0.830
MBOAT2 (Q6ZWT7)	0.820	OSBP (P22059)	0.819
FITM2 (Q8N6M3)	0.792	FASN (P49327)^a^	0.794
ABCA3 (Q99758)	0.801	PLA2G16 (P53816)	0.756
PNPLA8 (Q9NP80)	0.758	FABP5 (Q01469)	0.833
		LIPE (Q05469)^a^	0.578
		HADH (Q16836)	0.804
		AGPAT9 (Q53EU6)	0.833
		ALG10 (Q5I7T1)	0.540
		SLC27A3 (Q5K4L6)	0.803
		PTPLB (Q6Y1H2)	0.800
		ITPKC (Q96DU7)	0.793
		ABCA3 (Q99758)	0.587
		ELOVL5 (Q9NYP7)	0.702
		FADS3 (Q9Y5Q0)	0.781

^a^Validated by Western blot

## Discussion

The differentiation of MSCs into adipocytes is a complex process associated with diverse transcriptional changes [13]. Here, we identified a novel role for LRRC1 as a regulator of this adipogenic differentiation network.

We initially observed dynamic changes in LRRC1 expression levels in the context of adipogenesis. At present, the precise mechanisms governing LRRC1 expression are incompletely understood, with one study of hepatoma cells having shown decreased promoter methylation to contribute to the epigenetic upregulation of this gene in these cells [16]. In non-small cell lung cancer cells, however, LRRC1 expression was reported to be post-translationally regulated by miR-193a produced by bone marrow MSCs [17]. Here, we further found LRRC1 to be under the transcriptional control of PPARγ in the context of adipocytic differentiation. As PPARγ is a transcription factor that is essential to the regulation of adipogenesis, its knockdown can impair this physiological process [18–20]. Mechanistically, PPARγ binds to specific PPAR response element (PPRE) regions within target gene promoters to alter their expression [21]. Certain adipogenesis-associated genes are transcriptionally regulated by PPARγ, such as FATP (fatty acid transport protein) [22], adipocyte fatty acid binding protein (aP2) [23], and lipoprotein lipase (LPL) [24]. Notably, we herein found that while LRRC1 knockdown impaired adipocytic differentiation in MSCs, it had a negligible impact on PPARγ expression, suggesting a lack of feedback regulation between these two factors and underscoring LRRC1 as a secondary mediator of adipogenesis. Moreover, LRRC1 transcription is not solely regulated by PPARγ in this model system, as evidenced by the divergent expression patterns of these two genes on day 7 of the adipogenic process.

LRRC1 is a member of the LAP (leucine-rich repeat and PDZ) family of proteins that was initially identified as a regulator of cellular polarity, cell–cell connections, and oncogenic transformation [25]. Given that a loss of apical-basal polarity is generally related to malignant phenotypic outcomes in epithelial tissues, many studies have examined the oncogenic role of LRRC1. For example, in one report, LRRC1 was found to regulate breast cancer stem cell fate determination [11], while it has also been shown to influence HCC cell growth and colony formation [12], and to contribute to NSCLC cell cisplatin resistance [17]. LRRC1 also functions in non-oncogenic contexts, being expressed, for example, in myotubes wherein it influences the physical dimensions of agrin-dependent AChR aggregates and the density of microclusters formed in the absence of agrin [26]. Together with scribble and Erbin, LRRC1 also exhibits significant accumulation at neuromuscular junction (NMJ) regions in synaptic cells, likely regulating associated morphology and neurotransmission via nicotinic acetylcholine receptor clusters [27]. Moreover, LRRC1 is highly expressed in polarized epithelial tissue in *Xenopus laevis* embryos during the late stages of development, including the cement gland, eyes, tail bud, branch arcs, and developing otic vesicles [28]. These findings highlight the complex biological roles played by LRRC1.

At present, the signaling pathways engaged downstream of LRRC1 have yet to be fully clarified, although it has been shown to regulate WNT/β-catenin activity. Specifically, Scrib, a paralog of LRRC1, has been shown to negatively regulate WNT/β-catenin signaling in HEK293 cells [10]. Moreover, in LRRC1-knockout mice, LRRC1-deficiency induced higher levels of WNT ligand in breast cancer stem cells [11]. The WNT/β-catenin pathway serves as a key hub for the regulation of MSC adipogenic/osteogenic differentiation [29]. However, our data collected in the context of MSCs adipocytic differentiation did not provide any evidence for the ability of LRRC1 to regulate WNT/β-catenin signaling. This may be because we did not select sufficient detection time points. Instead, our proteomic analyses revealed significant changes in the expression of adipogenesis-related genes including fatty acid synthase (FASN, gene ID: 2194), hormone-sensitive lipase (LIPE, gene ID: 3991), and stearoyl-CoA desaturase (SCD, gene ID: 6319). FASN is a multifunctional enzyme responsible for catalyzing long-chain saturated fatty acid de novo biosyntehsis from acetyl CoA and malonyl COA when NADPH is available [30], with reduced FASN expression contributing to impaired adipogenesis [31]. LIPE can hydrolyze stored triglycerides in adipose and cardiac tissue to yield free fatty acids, with the dysregulation of its expression similarly contributing to aberrant adipogenic activity [32]. SCD is an iron-containing enzyme that is required for adipogenesis owing to its ability to catalyze a rate-limiting step in unsaturated fatty acid synthesis [33]. The functions of these three proteins are interrelated in the context of adipogenesis. However, the specific mechanisms whereby LRRC1 impacts the expression of these genes remains unclear and warrants further study.

In summary, these results support a model in which LRRC1 is a downstream PPARγ target that regulates the adipocytic differentiation of MSCs. Mechanistically, this regulatory activity may be associated with the control of the expression of adipogenesis-related proteins such as FASN, SCD, or LIPE. Together, these data enrich current understanding regarding the mechanistic basis for adipogenesis while providing a foundation for future functional studies of LRRC1.

## Supplementary Information

Below is the link to the electronic supplementary material.

Supplementary file1 (TIF 169 kb)—Fig. 1 Flow cytometry identification of the surface markers of sixth-generation hMSCs. A–H. Detection results for the hMSC surface markers CD14, CD34, CD45, HLA-DR, CD29, CD44, CD90, and CD105. I, J. Negative controls

Supplementary file2 (DOCX 12 kb)

Supplementary file3 (XLSX 131 kb)

## Data Availability

Enquiries about data availability should be directed to the authors.
