# Supplementary figures and images for: The downstream PPARγ target LRRC1 participates in early stage adipocytic differentiation
Source: Mol Cell Biochem. 2022 Nov 12;478(7):1465–73. doi: 10.1007/s11010-022-04609-8 (PMC10209303; doi:10.1007/s11010-022-04609-8)

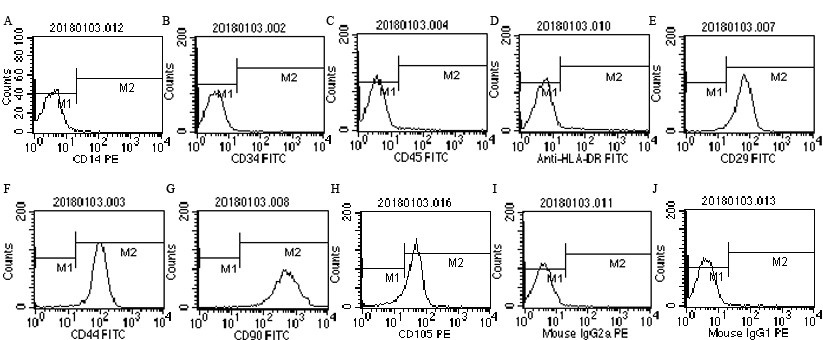

Supplement: Supplementary file 1 — Supplementary file1 (TIF 169 kb)—Fig. 1 Flow cytometry identification of the surface markers of sixth-generation hMSCs. A–H. Detection results for the hMSC surface markers CD14, CD34, CD45, HLA-DR, CD29, CD44, CD90, and CD105. I, J. Negative controls [file 11010_2022_4609_MOESM1_ESM.tif]
