# Supplementary material for: The downstream PPARγ target LRRC1 participates in early stage adipocytic differentiation
Source: Mol Cell Biochem. 2022 Nov 12;478(7):1465–73. doi: 10.1007/s11010-022-04609-8 (PMC10209303; doi:10.1007/s11010-022-04609-8)
Supplement: Supplementary file 2 — Supplementary file2 (DOCX 12 kb) [file 11010_2022_4609_MOESM2_ESM.docx]

Supplementary Table 1. Primer sequences used in PCR and the target sequence of shRNA in this manuscript.

|  | Forword 5’→3’ | Reverse 5’→3’ |
| --- | --- | --- |
| LRRC1 | CAGACTAACTCGGATACCTGCAG | CTGGTTGTCAGATAGCCACAGAG |
| PPARγ | AGCCTGCGAAAGCCTTTTGGTG | GGCTTCACATTCAGCAAACCTGG |
| CEBP/β | CAGGAGAAACTTTAGCGAGTCAGA | GGGTGGCCGCTATTAGTGAG |
| β-actin | GCGAGAAGATGACCCAGATCATGT | TACCCCTCGTAGATGGGCACA |
| ChIP primers for Binding site 1 | ATGTGCTCCCAAGTTACAAA | GGCGCTACTGGATTCGGTGT |
| ChIP primers for Binding site 2 | CAGGAGCTGTATGGAGGATG | TGCAGAAATAAGCAGGCAAA |
| Target interference for LRRC1 (1) | CATATCTTCCTGACTCTCTTA | |
| Target interference for LRRC1 (2) | GCAATCTTTATAACCTGGCTT | |
| Target interference for LRRC1 (3) | TTGCCAGAAAGCTTTCCTGAA | |
| Target interference for PPARγ | CAGCATTTCTACTCCACATTA | |
| Negative control Target sequence | TTCTCCGAACGTGTCACGT | |
